# Investigation on the Composition of Agarose–Collagen I Blended Hydrogels as Matrices for the Growth of Spheroids from Breast Cancer Cell Lines

**DOI:** 10.3390/pharmaceutics13070963

**Published:** 2021-06-26

**Authors:** Alessandra Quarta, Nunzia Gallo, Daniele Vergara, Luca Salvatore, Concetta Nobile, Andrea Ragusa, Antonio Gaballo

**Affiliations:** 1 CNR Nanotec, Institute of Nanotechnology, via Monteroni, 73100 Lecce, Italy; concetta.nobile@nanotec.cnr.it (C.N.); andrea.ragusa@unisalento.it (A.R.); 2 Department of Engineering for Innovation, University of Salento, via Monteroni, 73100 Lecce, Italy; nunzia.gallo@unisalento.it (N.G.); luca.salvatore@unisalento.it (L.S.); 3 Department of Biological and Environmental Sciences and Technologies, University of Salento, via Monteroni, 73100 Lecce, Italy; daniele.vergara@unisalento.it

**Keywords:** spheroids, tumoroids, hydrogel, collagen, agarose, mammary spheroids, tissue engineering, breast cancer, cisplatin, cancer therapy

## Abstract

Three-dimensional (3D) cell culture systems mimic the structural complexity of the tissue microenvironment and are gaining increasing importance as they resemble the extracellular matrix (ECM)–cell and cell–cell physical interactions occurring in vivo. Several scaffold-based culture systems have been already proposed as valuable tools for large-scale production of spheroids, but they often suffer of poor reproducibility or high costs of production. In this work, we present a reliable 3D culture system based on collagen I-blended agarose hydrogels and show how the variation in the agarose percentage affects the physical and mechanical properties of the resulting hydrogel. The influence of the different physical and mechanical properties of the blended hydrogels on the growth, size, morphology, and cell motility of the spheroids obtained by culturing three different breast cancer cell lines (MCF-7, MDA-MB-361, and MDA-MB-231) was also evaluated. As proof of concept, the cisplatin penetration and its cytotoxic effect on the tumor spheroids as function of the hydrogel stiffness were also investigated. Noteworthily, the possibility to recover the spheroids from the hydrogels for further processing and other biological studies has been considered. This feature, in addition to the ease of preparation, the lack of cross-linking chemistry and the high reproducibility, makes this hydrogel a reliable biomimetic matrix for the growth of 3D cell structures.

## 1. Introduction

Three-dimensional (3D) cell culture models, such as multicellular spheroids and organoids, have been demonstrated to mimic several biological processes in vitro much better than monolayer cell cultures [1,2]. There are essentially two methods to produce uniform-size multicellular spheroids: scaffold free, in which cells are prevented from adhering to the substrate but not to each other, thus forming spheroids, and scaffold- or matrix-based, in which cells are embedded into a three-dimensional biomaterial, such as a hydrogel [3]. The latter has the advantage of providing a physical structure comparable, in terms of stiffness and viscoelasticity, to the in vivo extracellular matrix (ECM), thus being able to reproduce not only cell–cell interactions, but also cell–matrix interplays directly related to phenomena associated with cell growth (cell fate), such as cytoskeletal organization, gene expression, nutrients diffusion, pH, and hypoxia [4]. This makes matrix-based spheroids extremely important for studying solid tumors microenvironment and their response to drug treatments [5].

Among 3D biomaterials, hydrogels of complex biological origin have been used in many studies [6,7,8]. The most common are Matrigel™, derived from Engelbreth-Holm-Swarm mouse tumor sarcoma, and other basement membrane-rich matrices. These hydrogels contain matrix membrane proteins, hormones, and soluble growth factors whose composition may vary among different batches. This aspect limits severely their use because, although they allow for the growth of spheroids, the batch-to-batch variability can alter cell culture systems and experimental results. Thus, simpler hydrogels, without hormones and growth factors, able to maximize reproducibility and offering the possibility to tune biochemical as well as mechanical properties, have been considered for growing spheroids. Hydrogels with these features are made of natural, synthetic, or hybrid materials, such as alginate, agarose, collagen, hyaluronic acid, and polyethylene glycol [1,9].

Among them, agarose is an inert, inexpensive, and easily available linear polysaccharide derived from red marine algae and consisting of alternating units of D-galactose and 3,6-anhydro-α-l-galactopyranose joined by α-1,3- and β-1,4-linkages. It possesses excellent biocompatibility, optimal gelling features, and tuneable mechanical properties that boosted its use as biomaterial for the manufacturing of tissue engineering scaffolds [10,11]. In addition, thanks to the ease of preparation, it has been recently exploited as non-adhesive and micromolded substrate for the growth of tumor spheroids based on multicellular aggregation [12,13]. Nevertheless, due to its poor bioadhesivity, its use as ECM-mimicking material is very limited.

On the other hand, type I collagen is the main protein component of the ECM in mammals. The presence of cellular binding sites (e.g., the “RGD” and “GxOGER” sequence, where “R” is arginine, “G” is glycine, “D” is aspartate, “O” is hydroxyproline, “E” is glutamate, and “x” is a hydrophobic amino acid) that promote cell adhesion, proliferation, and signalling makes collagen highly bioactive and suitable for the development of biocompatible hydrogels [14,15,16,17]. In addition, collagen plays a crucial role in tumor progression and invasion [18]. Nevertheless, it suffers of poor mechanical properties, limited stability over time, and high costs [19]. In this sense, blended hydrogels composed of agarose and collagen combine the mechanical properties of the former and the biomimetic nature of the latter.

As far as we know, only one study described the use of hydrogels containing agarose and collagen, as well as alginate, as matrices for producing tumor spheroids and their viability was monitored up to 14 days [20]. In addition, two studies described the use of agarose–collagen blended hydrogels to unravel the effects of biophysical cues on cellular mechanobiology of 2D intervertebral disc cells [21] and previously prepared glioblastoma spheroids [22]. Both works highlighted the crucial role of the hydrogel stiffness and adhesivity as driving forces that modulate the cell plasticity and connect the biological functionality to the surrounding physical stimuli. In living tissues, the stiffness ranges from few tens of Pa in intestinal mucus to GPa in bones, and variations of the mechanical properties are typically associated with cancer development and other diseases [23]. In the case of breast cancer, the stiffness has been shown to increase from hundreds of Pa to a few kPa when the normal tissue undergoes tumor transformation, due to the remodeling of the ECM [24].

Therefore, artificial matrices mimicking the physical properties of the naïve tumor environment can facilitate the study of the behaviour of 3D tumors in vitro and predict their response to modifications of the mechanical cues or to drug treatments, as it occurs during cancer progression and metastasis [4].

In this work, we developed collagen hydrogels blended with variable amounts of agarose (from 0.5% to 0.125%) and we investigated the possibility to grow within them tumor spheroids of three different breast cancer cell lines, i.e., the luminal estrogen receptor positive cells, MCF-7 and MDA-MB-361, and the triple negative model, MDA-MB-231. The physical properties of the matrices were analyzed in detail and the morphological characteristics of the spheroids were correlated over time to the hydrogel features. Preliminary drug testing studies with cisplatin and the possibility to recover the spheroids for additional studies were also evaluated.

## 2. Materials and Methods

Soluble type I collagen from calf skin was purchased from Symatese (Chaponost, France). Agarose, β-agarase from *Pseudomonas atlantica*, FITC-labeled hyaluronic acid, transferrin-TRITC, cisplatin, Dulbecco’s Modified Eagle Medium high glucose (DMEM), fetal bovine serum (FBS), penicillin and streptomycin were purchased from Sigma–Aldrich (Milan, Italy). Live/dead assay and MitoTracker Red CMXRos were purchased from Thermo Fisher (Rodano, Italy). Ultrapure grade water with a conductivity of 18.2 MΩ cm was used in all experiments. All chemicals were used as received.

### 2.1. Preparation of the Hydrogels

Agarose was dissolved in sterile phosphate-buffered saline (PBS, pH 7.4) to obtain a 1% (*w*/*v*) stock solution by heating on a hot plate. An aqueous suspension of 0.1% (*w*/*v*) type I collagen was obtained by slowly hydrating collagen flakes in distilled water for 3 h, under magnetic stirring at 4 °C. The blended hydrogels were prepared inside the wells of a 24 well plate, and each well was loaded to a final volume of 1 mL. In detail, the warm agarose solution (at around 45 °C, the gel point is at 36 °C) was added to the collagen suspension to obtain 3 different final concentrations, 0.5%, 0.25%, and 0.125%. The starting collagen solution was always diluted to 0.02% in the final blend. The mixture in each well was gently stirred with a glass rod and allowed to gel at room temperature. The gelation time varied from 30 s, in the case of hydrogels containing 0.5% agarose, up to 10 min, for those with 0.125% agarose. The gelation time was longer (up to 20 min) when the cells were embedded into the hydrogel, as all the preparation steps were carried out at 37 °C. All the glassware was sterilized in an autoclave before use, and the preparation of the hydrogels was carried out under a laminar flow hood. The preparation scheme is summarized in Figure S1.

### 2.2. Characterization of the Hydrogels

#### 2.2.1. Scanning Electron Microscopy (SEM)

Surface morphology and porosity of the hydrogels were investigated by using SEM imaging techniques. SEM images were recorded with a Carl Zeiss–Merlin field emission scanning electron microscope (Carl Zeiss, Oberkochen, Germany) equipped with a Gemini column and integrated with high efficiency in-lens and SE2 detectors, for high spatial/depth-of-field resolution secondary electrons (SE) imaging of surface structure and topography. The microscope was used in high vacuum and high-resolution acquisition mode and the images were recorded at an accelerating voltage of 5 kV and a few seconds frame-integration time, in order to minimize charging effects and sample damage. The hydrogels were cut, frozen, and lyophilized before imaging. The pore size was determined by measuring 50 random pores followed by statistical analysis using ImageJ software (NIH, Bethesda, MD, USA).

#### 2.2.2. Stability Test

The analysis was carried out keeping the hydrogels at 37 °C either in PBS or DMEM, and the samples were weighted at determined time points (up to 14 days). The stability of the hydrogels was then calculated as residual weight percentage:

$$Rw\%=\frac{w-w_{0}}{w_{0}}\times 100\%$$

where *w*_0_ is the starting weight of the hydrogel at *t* = 0, and *w* is the weight at each time point.

#### 2.2.3. Swelling Behavior

The swelling property of the hydrogels was determined by using a conventional gravimetric method [25]. Briefly, the dry weight of hydrogels was recorded before soaking them in PBS at 37 °C. The swelled weight of hydrogels was then measured at various time points, up to 48 h. The swelling behaviour was estimated as the percentage of the swelling ratio using the following equation:

$$Sr\%=\frac{w_{s}-w_{d}}{w_{d}}\times 100\%$$

where *w_d_* is the dry weight of the hydrogel and *w_s_* is the wet weight after hydration in PBS.

#### 2.2.4. Collagen Release

The agarose–collagen (A-C) hydrogels were kept in PBS at 37 °C and the volume of buffer (equal to 1 mL) was collected at determined time intervals to estimate the protein content via bicinchoninic acid (BCA) assay [26]. A calibration curve was set using collagen solutions at known concentrations. For each time interval, a blank sample (i.e., the PBS collected from agarose hydrogels prepared without collagen) was also measured.

#### 2.2.5. Fourier-Transform Infrared (FTIR) Spectroscopy

FT-IR spectra were recorded in transmittance mode on a Jasco 6300 spectrometer (Jasco Corp., Tokyo, Japan) between 4000 and 500 cm^−1^ with 40 scans and a resolution of 4 cm^−1^ and analyzed with the Spectra Manager software (Jasco). The measurements were performed by directly depositing the hydrogels onto the ATR crystal. The spectrum of each sample was acquired against a background obtained with the crystal without any sample. All analyses were carried out at room temperature.

#### 2.2.6. Mechanical Compression Test

The effect of the agarose concentration on the Young’s modulus of the A-C hydrogels was evaluated by uniaxial compression test. The correlation with time and temperature was also investigated. Briefly, the samples were incubated in PBS at 37 °C in a humidified atmosphere with 5% CO_2_. Cylinders of 8 mm were punched out and loaded into the testing chamber at fixed time points (0, 1, 4, and 8 days). All tests were performed with a universal ZwickiLine (Zwick Roell, Ulm, Germany) testing machine fitted with 10 N load cell. Loaded samples were hydrated in PBS at 37 °C and subjected to compression with a displacement rate of 0.01 mm/s, until 80% strain [27,28]. The compressive modulus was calculated by linear fitting between 2% and 10% of strain of the stress-strain curve. The test was performed in triplicate for each sample type and time point.

#### 2.2.7. Diffusion Test

PBS solutions containing either FITC-labeled hyaluronic acid (10 kDa molecular weight) or transferrin-TRITC (80 kDa molecular weight) at known concentration were prepared and deposited over the A-C hydrogels. The photoluminescence signal of the loaded solutions was recorded over time and the resulting concentration was extrapolated from the calibration curve of the corresponding standard staining solution. The diffusivity of the two molecules within the hydrogels was assessed determining the ratio between the concentration of the fluorescent molecule (either hyaluronic acid or transferrin) at the analyzed time point and that measured in free PBS.

#### 2.2.8. Drug Diffusion Test

1 mL of cisplatin solution (100 µM in PBS) was loaded above 1 mL of either 0.25–0.02% or 0.125–0.025% A-C hydrogels. Five time points (1, 2, 4, 8, and 24 h) were set and three duplicates for each time point and type of hydrogel were defined. The hydrogels were incubated at 37 °C. The cisplatin solution was withdrawn at the determined time points and the hydrogel was frozen before being lyophilized. Soon after, the samples were digested overnight in 65% nitric acid and the amount of Pt was estimated via elemental analysis using an Inductively Coupled Plasma Atomic Emission Spectrometer (ICP-AES) Varian 720-ES (Santa Clara, CA, USA).

### 2.3. Tumor Spheroid Preparation and Characterization

Human tumor cells were purchased from the American Type Culture Collection (ATCC). The human breast cancer cell lines, i.e., MCF-7, MDA-MB-361, and MDA-MB-231, and the human neuroblastoma SH-SY5Y cells were cultured as 2D monolayers in DMEM medium (4500 mg/L glucose) supplemented with 10% FBS, 100 U/mL penicillin, and 100 μg/mL streptomycin at 37 °C in an atmosphere of 5% CO_2_.

The cells were trypsinized, counted and added to the hydrogels at a density of 2.5 × 10^4^ cells/mL in complete growth medium. The cell suspension containing agarose and collagen was let to gel into the well plate. As an example, to prepare 1 mL of 0.25–0.02% A-C hydrogel, 250 µL of 1% agarose solution in PBS was added to 750 µL of a cellular suspension containing 200 µL of 0.1% collagen solution. After gelation, 1 mL of DMEM was added over the cell-embedded hydrogels before transferring the plate into the incubator. The medium was changed every 4 days.

#### Morphological Analysis of the Tumor Spheroids

The morphological characteristics of spheroids, including their diameter and shape, were determined by optical analysis using a EVOS XL Cell Imaging System microscope (Thermo Fisher, Waltham, MA, USA). The progressively developing spheroids were observed at 24 h intervals. The mean diameter of the 3D structures was calculated by using ImageJ Software (1.48 v).

For the SEM imaging, the tumor spheroids embedded within the hydrogels were fixed overnight with glutaraldehyde (2.5%) in cacodylate buffer (0.1 M) at 4 °C. The fixed specimens were washed three times with PBS and then 1% osmium tetroxide in a cacodylate buffer was added. After 6 h, the samples were washed three times with PBS, cut and lyophilized. Finally, the as-prepared samples were transferred to the SEM microscope for imaging. The operating conditions of the microscope were the same as those used for imaging the hydrogels.

### 2.4. Cellular Assays

#### 2.4.1. Live/Dead Assay

Viability of spheroids was investigated by using a live/dead assay kit (Thermo Fisher Scientific Inc., Waltham, MA, USA). Briefly, the activity of intracellular esterase induces non-fluorescent, cell-permeant calcein acetoxymethyl ester to become fluorescent after hydrolysis, giving a green fluorescence to the viable spheroids. On the other hand, ethidium homodimer enters and binds to nucleic acids only into damaged cells, producing a red fluorescence that indicates dead cells. The assay was performed at three time points to monitor the viability of the spheroids during their growth. In detail, the medium was removed from the plates containing the spheroids-embedded hydrogels and the samples were washed twice with PBS. A phosphate buffer solution containing calcein and ethidium homodimer was then added, and the plate was kept in incubation at 37 °C for 1 h. Finally, the solution was replaced with fresh PBS before imaging the samples under a Fluorescence Microscope (EVOS FLoid Cell Imaging Station, ThermoFisher, Waltham, MA, USA).

#### 2.4.2. Mitochondria Toxicity Assay

MitoTracker Red from Life Technologies was dissolved in PBS and added to the hydrogel containing the spheroids at the 5th day of growth (working concentration: 250 nM). The samples were kept in incubation for 1 h at 37 °C and then imaged under a Fluorescence Microscope (EVOS FLoid Cell Imaging Station, ThermoFisher, Waltham, MA, USA).

#### 2.4.3. Cisplatin Treatment

MCF-7 spheroids were grown in 0.25–0.02% and 0.125–0.02% A-C hydrogels up to 12 days. A DMSO solution of cisplatin was then added to the spheroids-embedded hydrogels to reach a final concentration of 100 µM. After 24 h incubation, the medium was removed and the hydrogels were carefully rinsed with fresh medium before performing the live/dead assay (ThermoFisher), as already reported.

### 2.5. Immunofluorescence Microscopy Analysis

After 5 days of growth within the hydrogels, the spheroids were fixed with ice-cold 4% paraformaldehyde for 30 min. Then, the spheroids were washed twice with PBS and incubated overnight with a mouse anti-E-cadherin (E-cad; Santa Cruz, sc-8426) antibody, according to the manufacturer’s protocol (1:1000 dilution in PBS). Subsequently, the samples were incubated with an anti-mouse Alexa Fluor 488 (AF488) conjugated secondary antibody (Cell Signaling). The images of fluorescently labeled proteins were captured using a fluorescence microscope (Leica LMD7000, Mannheim, Germany).

### 2.6. Enzymatic Digestion of Agarose for Spheroids Recovery

0.25–0.02% and 0.125–0.02% A-C hydrogels containing MCF-7 spheroids at different days of growth were incubated with 40 U of β-agarase from *Pseudomonas atlantica* and incubated overnight at 37 °C. The supernatant was then collected and observed under an optical microscope to visualize the presence of floating spheroids. Soon after, the spheroids were fixed with 4% paraformaldehyde and stained with 4′,6-diamidino-2-phenylindole (DAPI). The labeled structures were imaged under a fluorescence microscope (EVOS FLoid Cell Imaging Station, ThermoFisher, Waltham, MA, USA).

### 2.7. Ultrastructural Analysis of the Recovered Spheroids

The spheroids recovered from the hydrogels were fixed with glutaraldehyde (2.5%) in cacodylate buffer (0.1 M) at 4 °C for 1 h. The fixed specimens were washed three times with the same buffer, and 1% osmium tetroxide in a cacodylate buffer was added for 1 h. The cells were then washed again and dehydrated with 25%, 50%, 75%, and 100% acetone. Two steps of infiltration in a mixture of resin/acetone (1/1 and 2/1 ratios) were performed, and then the specimens were embedded in 100% resin at 60 °C for 48 h. Ultrathin sections (70 nm thick) were cut with an Ultramicrotome and then stained with lead citrate. TEM images were recorded on a JEOL Jem1011 microscope operating at an accelerating voltage of 100 kV (Tokyo, Japan).

### 2.8. Statistical Analysis

All data represent the average value of at least three independent experiments, unless otherwise specified. Normally distributed data was compared with a two-tailed Student’s *t*-test.

## 3. Results

### 3.1. Agarose–Collagen Hydrogels: Preparation and Characterization

Three agarose–collagen (A-C) hydrogels with different weight ratios were tested in this study. The collagen amount was kept constant to 0.02% in all the hydrogel formulations because it was already proven to be sufficient to provide enough anchoring sites to the embedded cells [22]. On the other hand, the amount of agarose, that confers the structural support, was varied from 0.5% to 0.125% to evaluate its impact on the biophysical properties of the hydrogel and, consequently, its effect on the cellular spheroids development. To prepare the hydrogels without denaturing the collagen and causing cell death, the agarose solution was first heated until boiling (100 °C) to dissolve the polysaccharide, and then cooled down to 45 °C. At this point, since gelation of pure agarose occurs at around 36 °C, it was rapidly mixed with collagen and the cell suspension (Figure S1).

**Figure 1:**
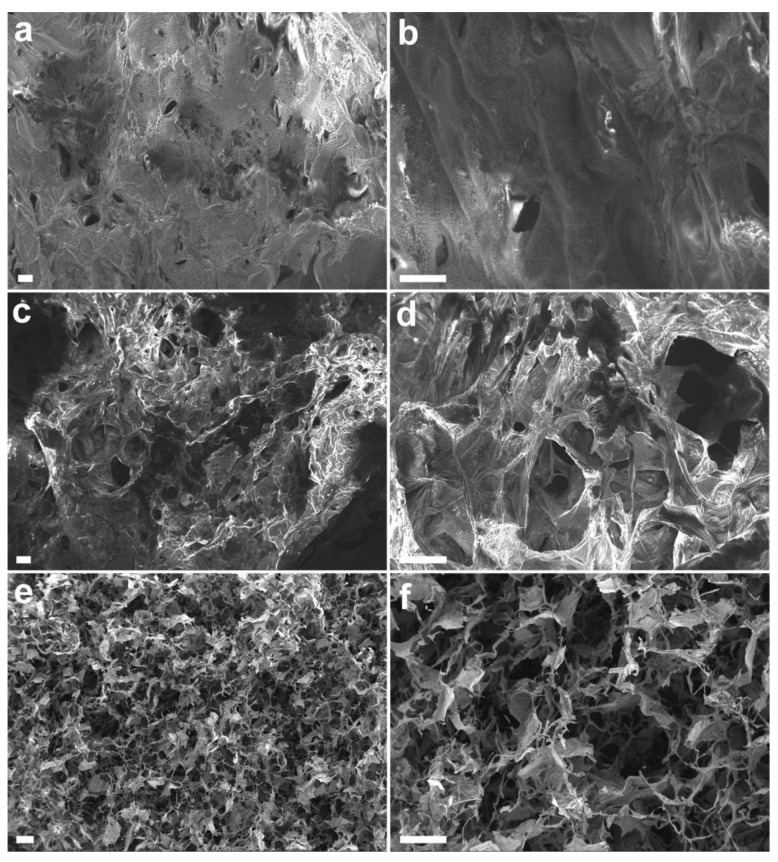
SEM images at lower (**a**,**c**,**e**) and higher (**b**,**d**,**f**) magnification of the A-C hydrogels with different agarose concentration: (**a**,**b**) 0.5%; (**c**,**d**) 0.25%; (**e**,**f**) 0.125%, respectively. Type I collagen is always 0.02%. Scale bar is 100 µm.

The SEM images of the resulting hydrogels showed that, by decreasing the agarose percentage, the porosity of the structure increases significantly, also appearing less compact (Figure 1).

In fact, the higher percentage of water in the 0.125–0.02% A-C hydrogel makes the dry structure very brittle, displaying pores with a mean size of 71 ± 14 µm that are quite uniform and interconnected (Figure 1e,f). Although the measure of the pore size of a dry structure cannot be considered realistic, it still provides a good estimation of the 3D organization of the hydrogels. The number of pores is smaller in the 0.25–0.02% and 0.5–0.02% A-C hydrogels, while the average pore size appears to be slightly larger (81 ± 21 and 87 ± 25 µm, respectively, Figure 1a–d). The higher turgidity and compactness of the hydrogels with higher percentage of agarose is also evident at a macroscopic level, where the 0.25–0.02% and 0.5–0.02% A-C hydrogels appeared self-standing, while that with 0.125% agarose did not (bottom panel of Figure S1).

**Figure 2:**
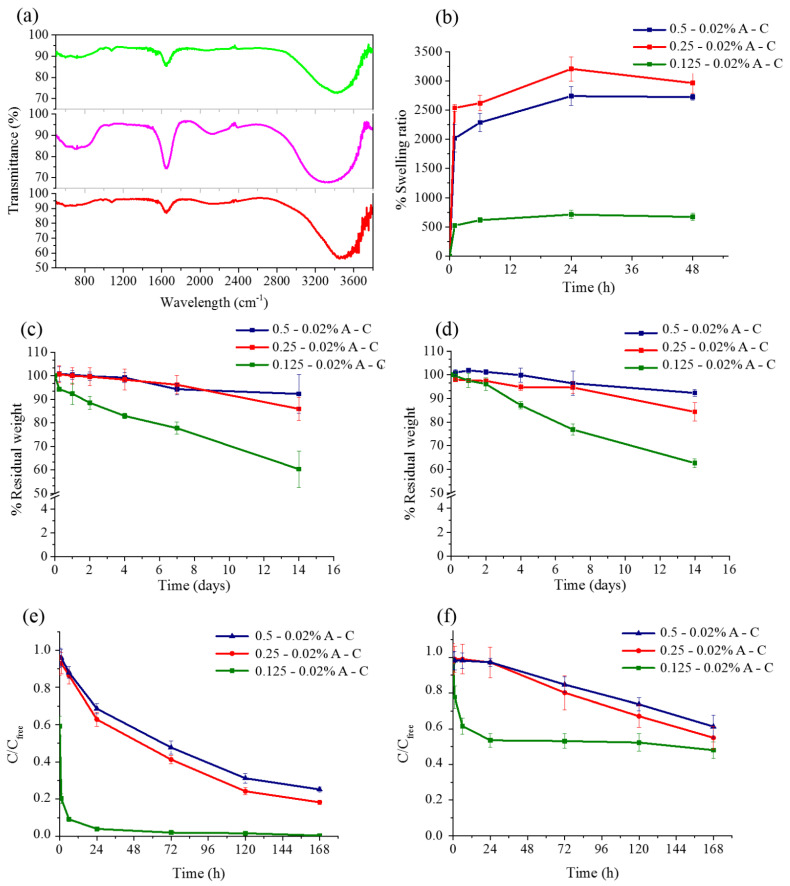
(**a**) FTIR spectra of agarose (green curve), collagen (pink curve), and 0.25–0.02% A-C (red curve) hydrogels. (**b**) Swelling behavior of the A-C hydrogels kept for 2 days in PBS at 37 °C. (**c**,**d**) Degradation curves up to 2 weeks of the A-C hydrogels either in PBS (**c**) or in DMEM (**d**). Diffusion tests performed up to one week with hyaluronic acid-FITC (**e**) and transferrin-TRITC (**f**).

FTIR spectroscopy was performed on the hydrogels to investigate whether collagen, despite the low percentage amount used, was detectable on the outer surface of the hydrogels. To this aim, the analysis was performed directly on the synthetized hydrogels deposited on the ATR crystal, without any further manipulation (Figure 2a).

Pure agarose hydrogel (0.25% *w*/*v*) was recorded as reference (light green line in Figure 2a), showing the typical signals at 988 and 1076 cm^−1^, relative to the C–H bending and to the C–O stretching of the glycosidic bonds, and two broad peaks at 1656 and 3421 cm^−1^, characteristic of the stretching of the H–O–H bound water and of the O–H hydrogen bonded carbohydrate hydroxylic groups, respectively [29]. Two broad peaks were also observed in the pure collagen hydrogel at about 3328 and 1645 cm^−1^ for the amide C=O and N–H stretching, respectively (pink line in Figure 2a). Additional smaller signals were detected at 1051 cm^−1^ for the C-OH stretching vibrations of carbohydrate moieties attached to the protein [30]. The FTIR spectrum of the blend 0.25–0.02% A-C hydrogel (red curve of Figure 2a) showed all the peaks characteristic of the pure compounds, i.e., smaller signals at 989 and 1079 cm^−1^, with a small side-bump at 1045 cm^−1^, and much broader peaks at 1649 and 3464 cm^−1^. Interestingly, while the last two peaks had similar intensity in pure collagen, a much higher intensity of the signal at lower frequencies was observed in pure agarose as well as in the blend hydrogel. As expected, no significant differences were observed in the spectra of the other two blend hydrogels (data not shown).

A critical feature of polymeric hydrogel is their capability to absorb and retain water, i.e., their swelling behavior. This property depends on many factors, such as network density, solvent used, and non-covalent interactions among all the components. In this case, the hydrogels containing 0.5% and 0.25% agarose showed a similar trend with a swelling ratio of about 20 and 25 times at t_0_ and a maximum swelling of about 27 and 30 times after 24 h in PBS, respectively (Figure 2b). These data are in accordance with those reported in the literature [31]. On the other hand, the ability to absorb water of the hydrogel with 0.125% agarose was considerably lower, with a swelling ratio of about 5 times at t_0_ and 7 after 24 h. Thus, there appears to be a critical threshold of agarose percentage below which the physical properties of the hydrogel are dramatically altered.

The stability over time of the three formulations was also investigated by measuring the percentage residual weight at 37 °C up to two weeks. The 0.5–0.02% and 0.25–0.02% A-C hydrogels were shown to be quite stable and lost around 10% and 15% of their weight after 14 days of incubation in PBS (Figure 2c) and DMEM (Figure 2d), respectively. On the other hand, the 0.125–0.02% A-C hydrogel showed a maximum of degradation close to 40% after 2 weeks in both incubation media. In parallel, the amount of collagen potentially released was estimated through the BCA assay (Figure S2). To this aim, the A-C hydrogels were kept in PBS at 37 °C and the volume of solvent collected and renewed every 24 h up to 14 days. Some collagen release was detected in all hydrogels, although to a higher extent and with a quicker trend in those with a lower agarose content. The overall percentage amount of collagen leaked after 2 weeks was equal to 50%, 27%, and 8% of the whole collagen present in the hydrogels containing 0.125%, 0.25%, and 0.5% agarose, respectively. This loss, together with the less compact texture of the 0.125–0.02% A-C hydrogel, would explain the higher degradation of this matrix.

A further parameter that deserves investigation is the capability of a hydrogel to allow the diffusion throughout the matrix of biomolecules and nutrients, such as growth factors and serum proteins having molecular weight of several tens of kDa. To this aim, a diffusion test was performed by using two fluorescent probes, FITC-conjugated hyaluronic acid and transferrin-TRITC, with a MW of around 10 and 80 kDa, respectively. Once loaded onto the three hydrogel formulations, the variation of concentration of the feeding solution was monitored over time and reported versus the diffusion in pure PBS. Hyaluronic acid diffused rapidly into the softest hydrogel, reaching 100% diffusion after 72 h (Figure 2e). The other two hydrogels performed similarly but with a more gradual diffusion that decreased by increasing the percentage of agarose, reaching a maximum of 82% and 75% after one week, respectively. On the other hand, transferrin-TRITC spread much more slowly, reaching after 24 h a diffusion value of almost 48% in the case of the 0.125–0.02% A-C hydrogel, while it was close to 10% in the case of the other two blends (Figure 2f). Noteworthily, after one week the diffusivity did not exceed 50% even in the softest matrix.

**Figure 3:**
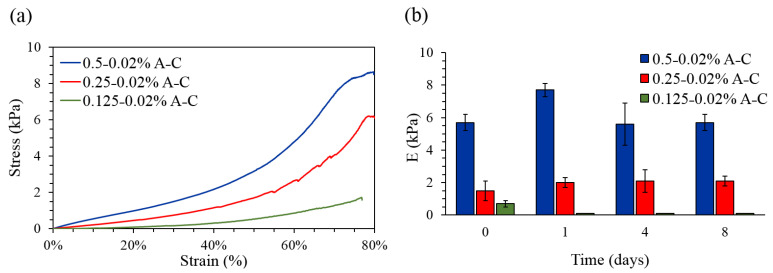
(**a**) Representative stress-strain curves of the A-C hydrogels subjected to unconfined compression with a displacement rate of 0.01 mm/s, until 80% strain. (**b**) Compressive moduli of the A-C hydrogels after 0, 1, 4, and 8 days of incubation in PBS at 37 °C, in humified atmosphere with 5% CO_2_.

The structural properties of the A-C samples were also evaluated by mean of compression test under physiological-like conditions showing how the agarose concentration deeply affects the hydrogels’ mechanical properties (Figure 3a).

In fact, an increase in the agarose concentration corresponded to an increase in the compressibility modulus. As expected, the 0.5–0.02% ratio showed the highest E modulus (5.7 ± 0.5 kPa), followed by the 0.25–0.02% (1.6 ± 0.4 kPa) (*p* = 0.0004) and the 0.125–0.02% (0.7 ± 0.2 kPa) (*p* = 0.0001) ones. A minor but still significant difference was found between the 0.25–0.02% and the 0.125–0.02% ratios (*p* = 0.03). In order to correlate degradation resistance to structural stability over time, the mechanical performances of the hydrogels were evaluated after 0, 1, 4, and 8 days of incubation in physiological-like conditions (PBS at pH 7.4, 37 °C, humified atmosphere with 5% CO_2_). While the 0.125–0.02% hydrogel could not be tested over time due to its low consistence, the 0.5–0.02% and the 0.25–0.02% blends retained their structural integrity until the 8th day of measure. As shown in Figure 3b, no significant changes in the E modulus were registered in the case of the 0.5–0.02% and the 0.25–0.02% hydrogels. These data are in accordance with those obtained by the degradation tests, in which a minimum weight loss was recorded.

### 3.2. Growth of Mammary Spheroids in A-C Hydrogels

One of the major advantages of hydrogels is their ability to provide more realistic 3D models for in vitro studies. In this study, we generated mammary spheroids from three different breast cancer cell lines, i.e., two luminal estrogen receptor positive cells (MCF-7 and MDA-MB-361) and a triple negative model (MDA-MB-231) for comparative analysis. The cells were seeded at the density of 2.5 × 10^4^ per mL inside the three types of hydrogels and time course studies (up to 14 days) were performed to monitor the process of multicellular spheroid formation. MCF-7 and MDA-MB-361 successfully formed spheroids in all the three types of hydrogels. On the other hand, MDA-MB-231 cells replicated during the first days, but they were not able to reach a defined 3D organization, thus not forming spheroids in any of the hydrogel conditions (Figure S3). It is worth to report that no multicellular spheroid formation was observed by culturing the three cell lines in 0.02% pure collagen, while the spheroids started to grow in the case of pure agarose hydrogel, but they underwent senescence after few days (data not shown).

**Figure 4:**
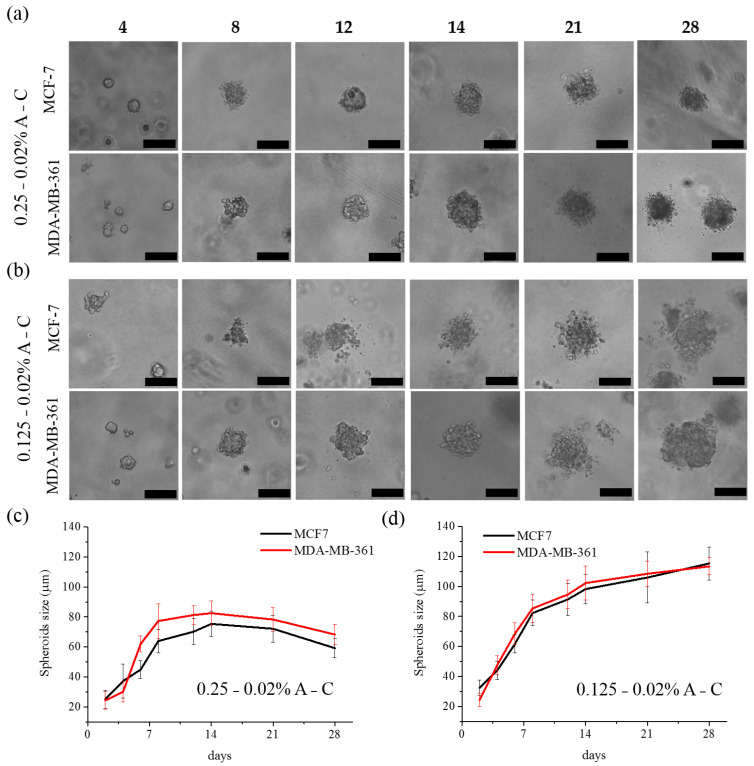
Optical images (**a**,**b**) and average size (**c**,**d**) of the spheroids obtained with MCF-7 and MDA-MB-361 cells grown up to 14 days either in 0.25–0.02% (**a**,**c**) or 0.125–0.02% (**b**,**d**) A-C hydrogels. Scale bar is 100 µm.

As reported, MCF-7 and MDA-MB-361 cells formed spheroids in the three blends, but the size and the morphology of the 3D structures were different depending on the experimental conditions. In particular, the spheroids grown in the stiffest hydrogel were spherical but smaller (Figure S4) than those grown in softer conditions (Figure 4), especially in the case of MDA-MB-361 cells.

This effect is more evident as the 3D structure progressively grows over time: the average size of MDA-MB-361 derived spheroids after 12 days grown in hydrogels with 0.5% agarose was around 63.1 ± 7.8 µm, while it reached 81.3 ± 6.3 µm and 94.7 ± 9.5 µm in 0.25–0.02% and 0.125–0.02% A-C hydrogels, respectively (Table S1). In the case of MCF-7 cells, the size gap was of about 7 and 27 µm respectively, being the spheroids in the hydrogels with 0.5% agarose about 64 µm large after 12 days, while they reached a diameter of about 70 µm in the 0.25–0.02% A-C hydrogel and about 91 µm in the softest one (Table S1). The images in Figure 4 clearly evidence how the 3D structures evolved over time from a single cell to more complex aggregates, but the growth curves showed that while the spheroids in the hydrogel with 0.25% agarose reached a growth plateau after 14 days, they still displayed a positive growth trend in the softest environment. In fact, the spheroids in the softest matrix continued to grow exceeding the 100 µm diameter in both cell lines after 28 days. On the other hand, the spheroids in the 0.25–0.02% A-C hydrogel stopped their growth and, after 2 weeks, they started to shrink and exhibited a dark intracellular substance. In this sense, it appears that the softest hydrogel is capable of sustaining the spheroids’ growth for longer time although, especially in the case of MCF-7, they displayed irregular contours and looser structure [32,33]. As a general consideration, we observed that MCF-7 cells could tolerate stiffer environments as compared to MDA-MB-361, as confirmed by the fact that they also generated small spheroids in hydrogels with 1% agarose (Figure S5), while MDA-MB-361 did not.

**Figure 5:**
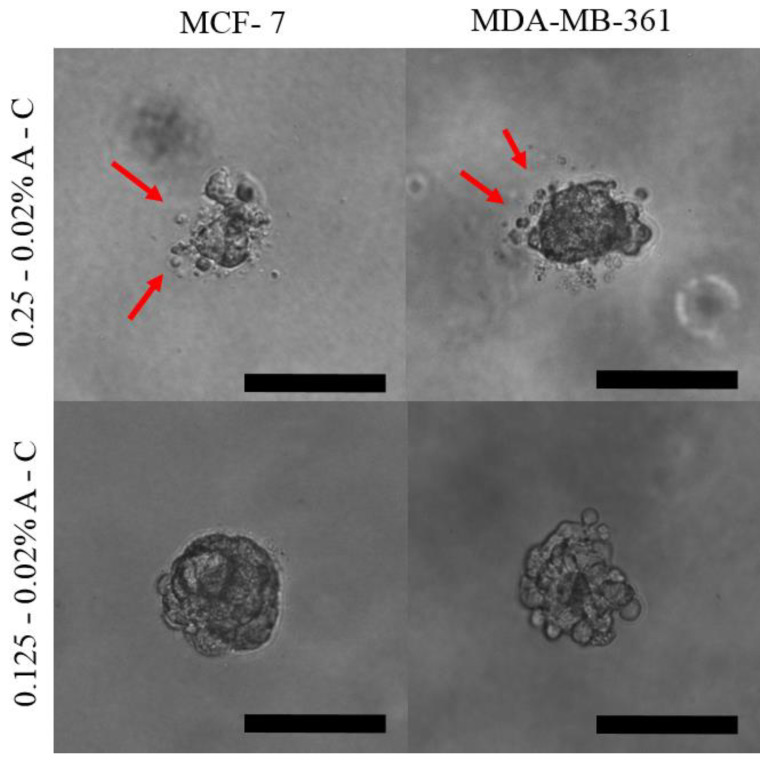
Optical images of the tumor spheroids obtained by MCF-7 and MDA-MB-361 cells after growing for 8 days in either 0.125–0.02% (**top**) or 0.25–0.02% A-C hydrogels (**bottom**). The red arrows point to the cells disseminated by the spheroids grown in the soft matrix. Scale bar is 100 µm.

In conclusion, the hydrogel stiffness and the matrix composition regulated the spheroids growth and morphology and, more interestingly, they affected the local migration of the outer cells. In fact, only the softest matrix was able to induce protrusion of cells from the outer layer and their local dissemination (Figure 5).

As already stated, the motility of these cells depends on the interaction with the microenvironment, mainly with collagen [21,34]. It is likely that the less dense hydrogel facilitates the protrusive behavior, also facilitating contact with the collagen anchoring points for the spatial dissemination of the cells. On the other hand, the reduced migration of the outer cells in the hydrogels with 0.25% agarose should be related to the tighter pressure that the matrix exerts on the cells, leading, as already described, to more compact spheroids.

Based on these observations and with the aim to study the effects of the mechanical features of the environment on the tumoroids features, the following analyses were carried out comparing the two conditions (0.25% and 0.125% agarose-based hydrogels) in which both cell lines were able to form healthy and stable spheroids up to two weeks.

### 3.3. Mammary Spheroids Viability and Epithelial Markers Expression

**Figure 6:**
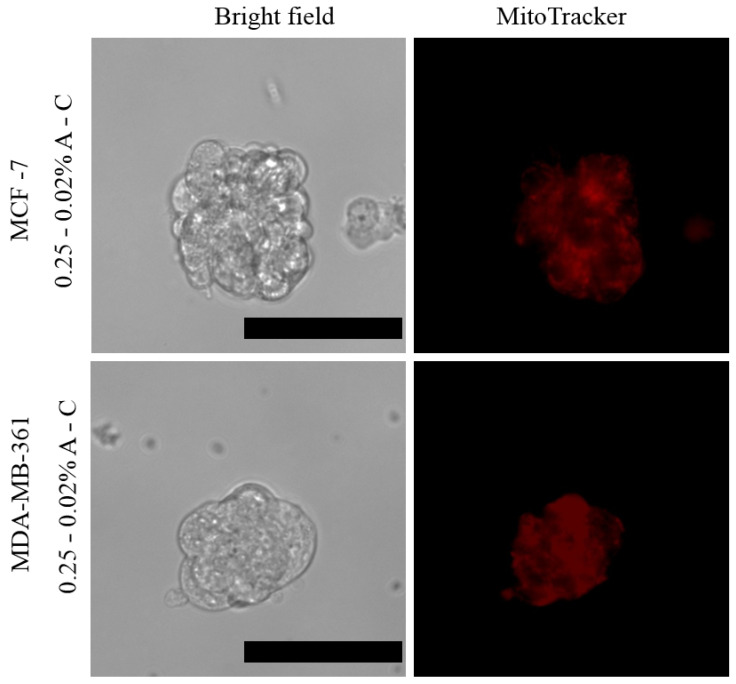
Mitochondrial labeling with MitoTracker red of living spheroids of MCF-7 and MBA-MB-361 cells after 5 days of growth. Scale bar is 50 µm.

The viability of the spheroids obtained from the MCF-7 and MDA-MB-361 cell lines in both type of hydrogels was investigated through a live/dead fluorescence assay (Figure S6). The homogeneous green fluorescence evidenced that all the cells in the spheroids were viable after 8 days; while few red spots were already visible after 14 days in the 3D structure grown in the matrix with 0.25% agarose, showing their initial aging. On the other hand, the spheroids embedded in the 0.125–0.02% A-C hydrogel resulted absolutely viable. These data are in accordance with the previous analysis of the growth curves of the 3D structures in the two systems (Figure 4). To further confirm this trend, the assay was also performed after 28 days. Notably, while the spheroids grown in the 0.125% agarose-based hydrogels were still viable, those prepared in the stiffer matrix were dead. Furthermore, MitoTracker red, an indicator of mitochondrial membrane potential able to selectively stain active mitochondria, was used to gain information on the mitochondrial function (Figure 6).

MitoTracker staining showed the presence of active mitochondria in the periphery as well as in the center of the spheroids.

**Figure 7:**
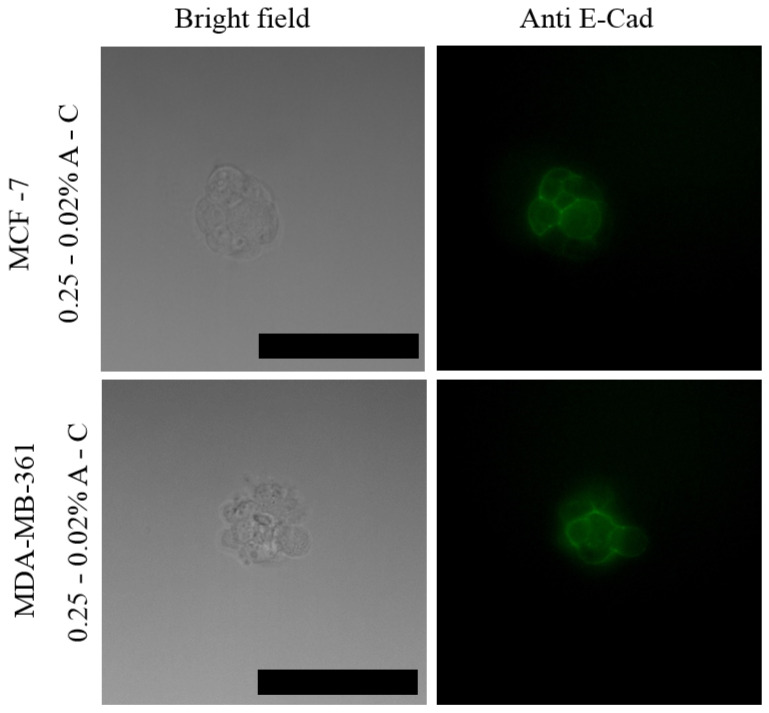
Staining of E-cadherin in cellular spheroids of MCF-7 and MBA-MB-361 after 5 days of growth in A-C hydrogels. Scale bars correspond to 58 µm.

The expression of E-cadherin, a typical epithelial marker in 3D spheroids, was then determined by immunofluorescence, clearly showing that the mammary spheroids maintained the expression of the transmembrane glycoprotein and evidencing the presence of tight cell–cell interactions, both typical features of an epithelial phenotype (Figure 7). Similar results were obtained with the 0.125–0.02% A-C hydrogel for both MitoTracker and E-cadherin staining (data not shown).

The morphology of the MCF-7 spheroids grown for 12 days and their arrangement into the 0.25–0.02% A-C hydrogel was also investigated by ultrastructural analysis, showing a 3D structure wrapped into the hydrogel matrix (Figure S7). The protrusion of the spheroids from the hydrogel can be clearly appreciated and they can be compared to cocoons anchored to a branch.

Overall, these data suggest that this type of hydrogel is a suitable approach for the generation and growth of mammary spheroids.

### 3.4. Cisplatin Delivery to the Embedded Spheroids

**Figure 8:**
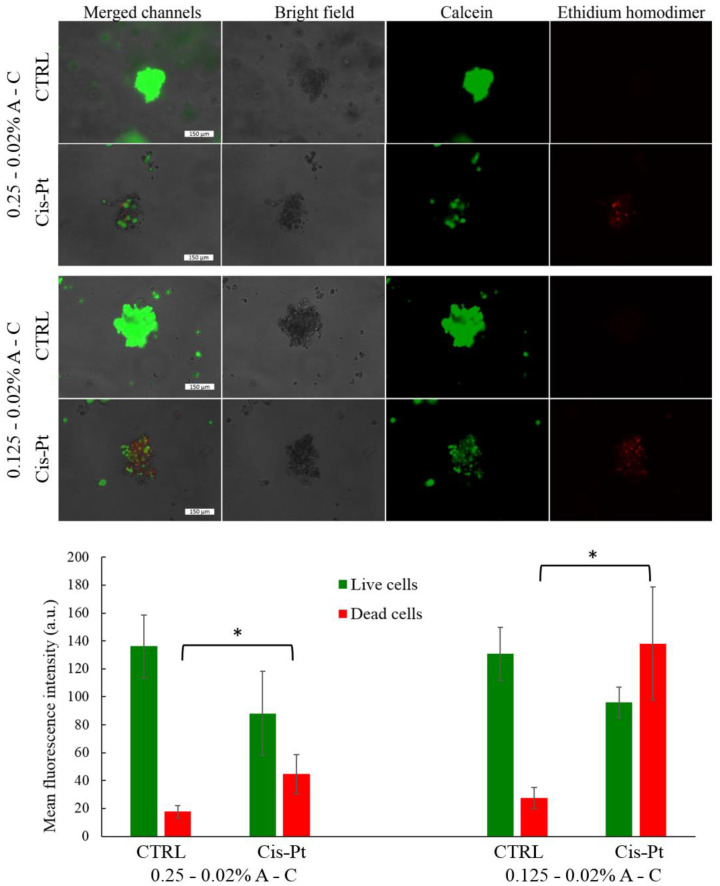
Live/dead assay performed with the MCF-7 spheroids embedded into the 0.25–0.02% (**a**) and 0.125–0.02% A-C hydrogels (**b**) after 24 h incubation with 100 µM cisplatin. (**c**) Mean fluorescent intensity detected in the 0.25–0.02% (left) and 0.125–0.02% (right) A-C hydrogels. Green bars correspond to the fluorescence signal of calcein, while red bars to ethidium homodimer, respectively. (* indicates *p* < 0.01 when comparing CTRL spheroids with cisplatin-treated spheroids in both types of hydrogel. Statistical significance was assessed by *t*-test).

To evaluate the exploitation of these 3D systems as a drug-screening platform, the MCF-7 spheroids embedded either in the 0.25–0.02% or 0.125–0.02% A-C hydrogels were treated with 100 µM cisplatin. This concentration was chosen in accordance with recently published studies in which the drug response of hydrogel-embedded spheroids was assayed [35,36,37]. In a preliminary experiment the diffusion time of cisplatin into the hydrogels (without the spheroids) was determined via elemental analysis. As shown in Figure S8, the drug diffusion was faster in the softer hydrogel, reaching a 100% rate (concentration at the equilibrium) already after 2 h incubation at 37 °C. In the case of the 0.25–0.02% A-C hydrogel, the maximum diffusion was detected after 8 h of incubation. Based on these findings the incubation time of the spheroids with the drug was set at 24 h. After drug treatment, the cell mortality was estimated by the live/dead assay: dead cells were detected in the hydrogels administered with cisplatin, while control samples were brightly green fluorescent (Figure 8).

The analysis of the distribution of the fluorescent pixels performed on 25 spheroids for each type of sample evidenced that the difference between the number of dead cells of the control and those of the drug-treated samples is statistically significant (*p* < 0.01) (Figure 8, lower panel). This assay confirmed that the drug diffusion depends on the agarose percentage amount, as the number of dead cells was higher in the hydrogel containing 0.125% agarose. Interestingly, the structure of the spheroids was dramatically altered if the samples, after 24 h incubation with the drug, were kept in fresh medium for additional 5 days. A large number of dead cells detached from the spheroids and many cellular debris were scattered in the matrix, while small residues of the 3D structures were still visible (Figure S9). This effect was observed in both types of hydrogels after 5 days post drug treatment.

### 3.5. Enzymatic Digestion of Agarose for Spheroids Recovery

**Figure 9:**
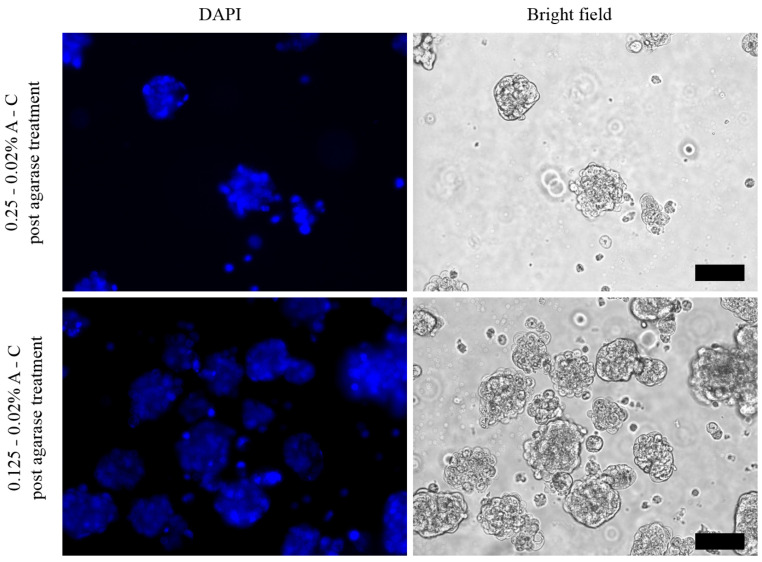
Optical images of MCF-7 spheroids grown in the 0.25–0.02% (upper panels) and 0.125–0.02% (lower panels) A-C hydrogels, recovered after agarase treatment, fixed and stained with DAPI. Scale bars correspond to 100 µm.

To evaluate the possibility to recover the spheroids from the hydrogels for additional processing and/or other biological studies, the blends with 0.25% and 0.125% agarose containing MCF-7 spheroids at different days of growth were incubated with β-agarase from *Pseudomonas atlantica* [38,39]. However, only the spheroids located in the outer layer of the 0.25–0.02% A-C hydrogel were recovered after overnight incubation at 37 °C with the enzyme (upper panels in Figure S10). Most of the hydrogel remained intact and the spheroids continued to grow inside it as in control hydrogels not treated with agarase. On the other hand, the overnight enzymatic treatment completely dissolved the hydrogel containing 0.125% agarose, and all the spheroids could be recovered (lower panels of Figure S10). Noteworthily, the stability test (Figure 2) already showed a higher degree of spontaneous degradation of the hydrogel with 0.125% agarose compared to that higher amount. Thus, the addition of agarase boosted the degradation process, leading to the complete dissolution of the softer matrix. The morphology of the spheroids collected from both types of hydrogels after agarase treatment was preserved even at different times of growth, up to 12 days (Figure S10). The vitality of the recovered spheroids was also confirmed by the live/dead assay (Figure S11) as well as by DAPI staining (Figure 9), showing their suitability for further processing and study. Only a few free individual cells could be observed, probably detached from the surface of the spheroids during the centrifugation steps required by the staining protocol.

Nevertheless, the spheroids preserved their shape, morphology, and vitality characteristics, confirming their suitability for subsequent biological studies (Figure S11).

As proof of concept of the applicative potential of the recovered spheroids, they were processed for ultrastructural imaging. The TEM images of Figure S12 show neighbouring cells tightly connected through cellular junctions and cellular organelles typical of metabolically active cells.

## 4. Discussion

In this work blended hydrogels composed of agarose (variable weight amount from 0.125% to 0.5%) and collagen (fixed weight amount equal to 0.02%) were prepared as enabling matrices for the growth of 3D cellular structures. These hydrogels combine the biomechanical properties of agarose and the bioadhesivity of collagen. The amount of collagen is 6.25-, 12.5-, and 25-times lower than that of agarose, and the formation of the hydrogel is probably to be ascribed mainly to agarose. In fact, although the self-assembling capability of collagen molecules in vitro under physiological conditions is well known, reconstituted collagen fibrils, that are held together by non-covalent interactions (hydrogen bonding, hydrophobic and electrostatic interactions) are free to slide and do not form a stable 3D network [40,41,42]. In addition, in preliminary experiments we have observed that collagen itself does not go into the gel state under the same experimental conditions we used to obtain the A-C hydrogels (data not shown). Thus, the gelation likely depends upon the formation of intra- and inter-molecular hydrogen bonds in the agarose backbone at a temperature lower than <40 °C, as elsewhere reported [43].

Although agarose and collagen have already been used to prepare hydrogels, they still represent valuable candidates for creating and exploiting viable hydrogels because of their biocompatibility and low cost. More complex hydrogel composed of expensive polymers have been also developed for growing spheroids, but they suffer of limited availability and high costs of the products. Furthermore, it is worth reminding that agarose gelation does not require chemical crosslinking (whose residues would have effects on cell viability) and occurs at temperatures compatible with cell growing conditions.

Here we suppose that the A-C hydrogel formulations combine the mechanical support for 3D cell growth on the one hand, and the biomimetic component on the other. In this sense, agarose acts as the structural backbone of the matrix while collagen provides biological fingerprints for the growing 3D structures [20,32].

As expected, the characterization of the hydrogels evidenced that the agarose percentage governs the morphological, structural, and mechanical features of the matrix. Reasonably, the greater the amount of agarose, the stiffer the hydrogel resulted. On the other hand, the lowest agarose amount corresponded to the fastest hydrogel degradation and diffusion of the molecules through the matrix. The higher degree of degradation of the softest hydrogel was also associated to a higher release of collagen. Thus, it can be expected that a less compact hydrogel facilitates the release of collagen, as determined by the protein quantification assay.

Similarly, the entry and movement of biomolecules through the blended hydrogels appears to be associated to the agarose percentage. The bigger the biomolecule and the stronger the type of non-covalent interactions it can establish with the matrix, the slower they are and the lesser the total amount of molecules that may reach the cells embedded into the matrix. In this sense, the hydrogel could act as a physical barrier to the diffusive transport of specific nutrients and drugs to the spheroid, similarly to what occurs in vivo [44].

The hydrogels were exploited for growing tumoroids from three breast cancer cell lines, namely MCF-7, MDA-MB-361, and MDA-MB-231 cells. However, while MCF-7 and MDA-MB-361 cells formed nice 3D structures, MDA-MB-231 cells, a triple negative breast cell line, did not organize into any three-dimensional arrangement, in accordance with previous findings [45,46], probably because of their lack of adherens junctions.

The spheroids generated by MCF-7 and MDA-MB-361 cells presented size and compactness strictly related to the stiffness of the surrounding matrix, and thus to the percentage of agarose. Apparently, both breast cancer cells prefer hydrogels with a stiffness from about 1.5 to 0.7 kPa, i.e., with a percentage of agarose equal or below 0.25%, while stiffer matrices, such as that with 0.5% agarose, did not result suitable to support the growth of the spheroids. A different tissue-specific tropism of the cellular models probably contributes to this result. In fact, MCF-7 cells have a low metastatic potential and are not tissue-specific, while MDA-MB-361 cells were derived from a brain metastasis and their growth on a softer hydrogel could match their in vivo metastatic microenvironment. With the intent to partially confirm this hypothesis, the 3D growth of a neural cell model was examined. SH-SY5Y neuroblastoma cells generated spheroids in the 0.125–0.02% A-C hydrogel, while in the stiffer matrix after a slow cellular duplication, the structures did not grow further over time (Figure S13). As a general remark, SH-SY5Y spheroids reached larger diameters (121.7 ± 14.2 µm) than those obtained with MCF-7 and MDA-MB-361 cells grown for 14 days in the same matrix (i.e., 98.3 ± 10.0 and 101.3 ± 11.4 µm, respectively), and this heterogeneity could be probably related to the different origin of the cell line.

The live/dead assay and the mitochondrial staining showed that the spheroids are viable up to 14 days in both types of hydrogels. Notably, the 3D cultures were monitored for up to one-month, evidencing a continued growth of the spheroids in the softer hydrogel, reaching an average size larger than 100 µm. On the other hand, the spheroids in the 0.25–0.02% A-C hydrogel stopped their growth after 2 weeks, resulting completely dead after 4 weeks. This different behavior might be related to the tighter interactions between the cells and the surrounding environment as the 3D structures grow over time, i.e., the limited degradation and capability of the stiffer hydrogel to accommodate the spheroids induced their slow aging and death.

These results confirm that soft agarose–collagen hydrogels allow for long-term spheroid growth although cells derived from different tissues sense the change in stiffness of the substrate and significantly modify their behavior. In this respect, it has been shown that cells respond to ECM environment by regulating a plethora of transcription factors and other signals that affect cytoskeleton, cellular uptake, and cell cycle, that in turn determine their morphology, proliferation, differentiation, tumor invasion and metastasis [47,48,49,50]. Among them, it is worth mentioning the transmembrane glycoprotein E-cadherin that is expressed in epithelial cells and connect them through lateral adherent junctions. It has been demonstrated that the level of expression of E-cadherin represents a crucial feature in cancer progression as it is involved in the epithelial-mesenchymal transition [51]. Loss of E-cadherin expression is generally associated to a lack of intercellular contacts and to an increased tumor cell invasiveness through the activation of signaling pathways that regulate metastatic progression [52]. E-cadherin expression is also associated to the formation of multicellular tumor spheroids, as already demonstrated [1]. Expression of E-cadherin was also observed in the spheroids derived from MCF-7 and MDA-MB-361 cells, as shown in Figure 7, confirming its importance in tumor development and progression, and the suitability of these spheroids for mimicking natural tissues. This was also demonstrated by successfully delivering a common chemotherapy drug, i.e., cisplatin, through the matrix.

In conclusion, as a general consideration from all the experiments performed, softer hydrogels allowed for the establishment of long-term culture of large and irregular breast tumor spheroids, while stiffer environmental conditions favored the growth of small and compact 3D cell strictures viable for shorter periods (Figure S14). Finally, the possibility to recover the tumoroids was demonstrated by treating the hydrogels with agarase. The yield of the recovery process was quantitative and highly reproducible in the softest gel, that already underwent partial spontaneous degradation, while in the matrix with 0.25% agarose only a small percentage of 3D structures could be successfully recovered for additional biological and biomolecular studies.

## 5. Conclusions

A simple (no crosslinking steps are required), cost-effective, and highly reproducible method for the generation of tumor spheroids has been reported. The optical transparency of the matrix facilitates the daily monitoring of the cellular growth and the morphological variations due to drug testing. The procedure consists in wrapping individual tumor cells in agarose hydrogels (with either 0.25% or 0.125% weight percentage) blended with 0.02% collagen, where agarose reproduces the biomechanical features of the ECM, while collagen provides the anchoring sites for the membrane proteins. The growth of tumoroids deriving from three breast cancer cells lines was investigated in detail and compared. Interestingly, one cell line, MDA-MB-231 did not form spheroids in any of the conditions employed, while the other two lines, MCF-7 and MDA-MD-361, displayed quite similar behavior.

The variation in the agarose amount affected the physical and mechanical features of the resulting hydrogel and the growth of the spheroids. In fact, the stiffer the hydrogel, the more compact and slightly smaller the tumoroids resulted. Therefore, depending on the type of tumoroids to be prepared and studied, the composition of the hydrogel can be easily tuned. The growth of the spheroids was monitored for up to 2 weeks and the qualitative analysis of their viability evidenced few dead cells only after 14 days.

Preliminary studies of drug testing with cisplatin showed that the blended hydrogels allow for tracking the response of the spheroids to the drug administration. This aspect makes the system potentially useful for routine drug screening.

Finally, the degradability of the hydrogels upon enzymatic treatment was demonstrated, leading to complete recovery of the tumoroids in the case of the softest hydrogels, while to a partial recovery in the hydrogel with 0.25% agarose. The possibility to recover the spheroids is of a paramount importance as it enables further biomolecular studies on the collected samples, showing the full potential of this cheap and easily scalable biomimetic hydrogel.

## Supplementary Materials

The following are available online at https://www.mdpi.com/article/10.3390/pharmaceutics13070963/s1, Figure S1: schematic drawing of hydrogels’ preparation and pictures of the obtained hydrogels, Figure S2: BCA assay, Figure S3: optical images of MDA-MB-231 spheroids grown in the 0.25–0.02% A-C hydrogel after 2, 4, 6, and 8 days, Figure S4: optical images of MCF-7 and MDA-MB-361 spheroids grown in the 0.5–0.02% A-C hydrogel after 6, 8, and 12 days, Table S1: average size of the tumor spheroids after 12 days, Figure S5: optical images of MCF-7 spheroids grown in the 1–0.02% A-C hydrogel after 6, 8, and 12 days, Figure S6: live/dead assay performed with the cellular spheroids embedded into the A-C hydrogels at 8, 14, and 28 days, Figure S7: SEM images of MCF-7 spheroids embedded in the 0.25–0.02% A-C hydrogel after 12 days of growth, Figure S8: cisplatin diffusion in 0.25–0.02% and 0.125–0.02% A-C hydrogels estimated via elemental analysis, Figure S9: optical images of MCF-7 spheroids grown in 0.125–0.02% A-C hydrogel after 24 h cisplatin treatment and additional 5 days of incubation with fresh medium, Figure S10: optical images of MCF-7 spheroids grown in 0.25–0.02% and 0.125–0.02% A-C hydrogel and recovered after incubation of the hydrogels with agarose, Figure S11: live/dead assay performed with the MCF-7 spheroids grown for 8 days in the 0.125–0.02% A-C hydrogel and recovered after agarase treatment, Figure S12: a–d) TEM images of MCF-7 spheroids grown for 8 days in the 0.125–0.02% A-C hydrogel, Figure S13: optical images of SH-SY5Y spheroids grown in the 0.125–0.02% and 0.25–0.02% A-C hydrogels up to 14 and 6 days, respectively, Figure S14: sketch depicting the effects over time of the hydrogel stiffness on the size and viability of breast tumor spheroids derived from MCF-7 and MDA-MB-361 cells.
