# Supplementary material for: Investigation on the Composition of Agarose–Collagen I Blended Hydrogels as Matrices for the Growth of Spheroids from Breast Cancer Cell Lines
Source: Pharmaceutics. 2021 Jun 26;13(7):963. doi: 10.3390/pharmaceutics13070963 (PMC8308953; doi:10.3390/pharmaceutics13070963)
Supplement: Supplementary file 1 [file pharmaceutics-13-00963-s001.zip › pharmaceutics-1247446-supplementary.pdf]

pharmaceutics

MDPI

Supplementary Materials: Investigation on the Composition of Agarose–Collagen I Blended Hydrogels as Matrices for the Growth of Spheroids from Breast Cancer Cell Lines

Alessandra Quarta, Nunzia Gallo, Daniele Vergara, Luca Salvatore, Concetta Nobile, Andrea Ragusa and Antonio Gaballo

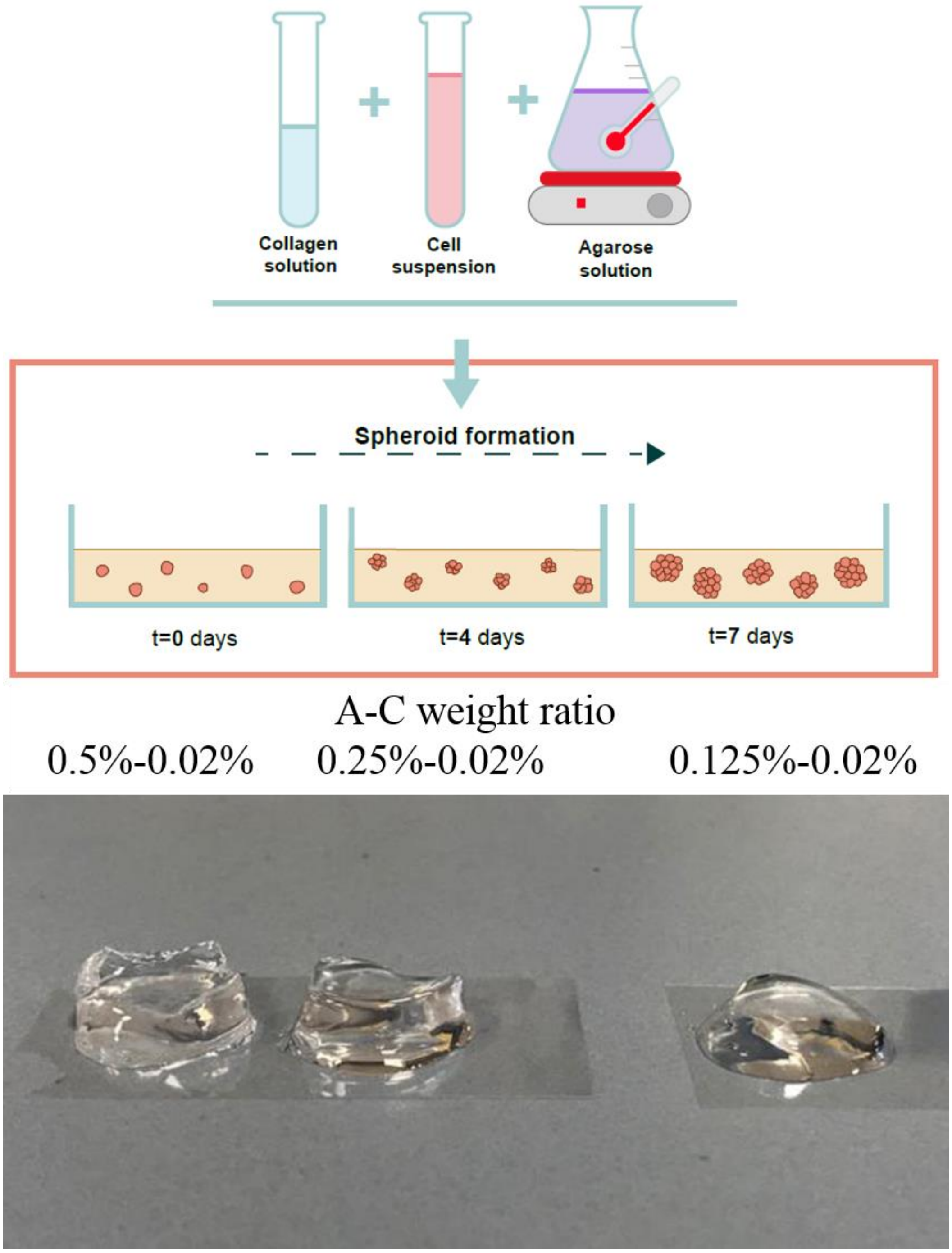

Figure S1. Schematic drawing showing the preparation of the A-C hydrogel containing tumour cells and subsequent spheroids formation (top). Image of the obtained A-C hydrogels (bottom).

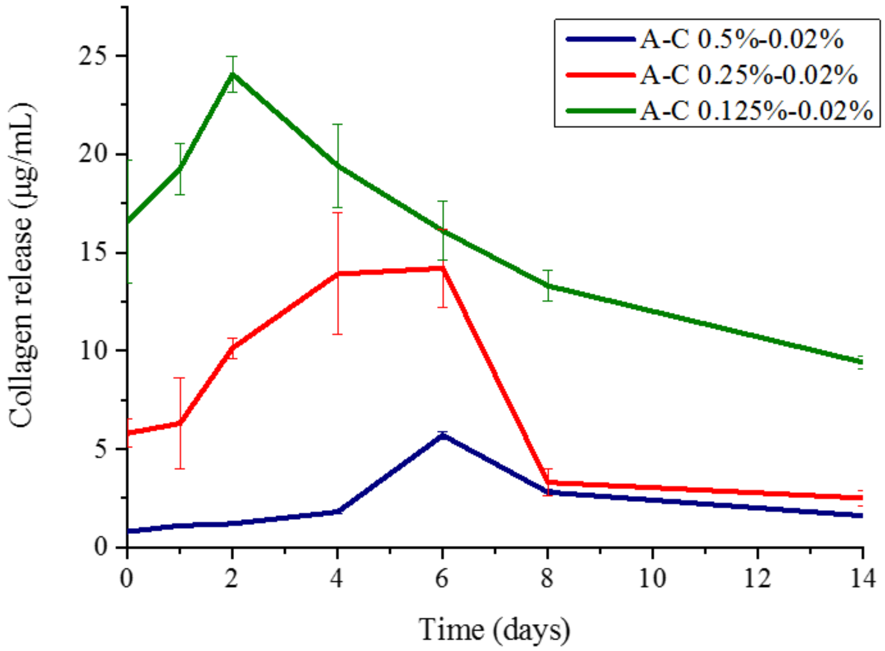

Figure S2. BCA assay performed with A-C hydrogels at three weight ratios and at six time points up to 14 days. Time point 0 corresponds to 3 h after preparation.

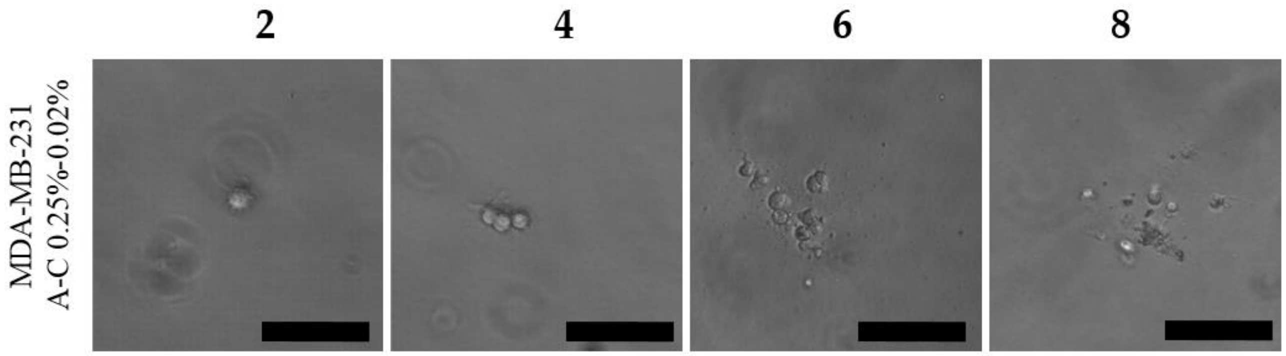

Figure S3. Optical images of MDA-MB-231 cells grown in the 0.25–0.02% A-C hydrogel after 2, 4, 6, and 8 days. Scale bar is 100 μm.

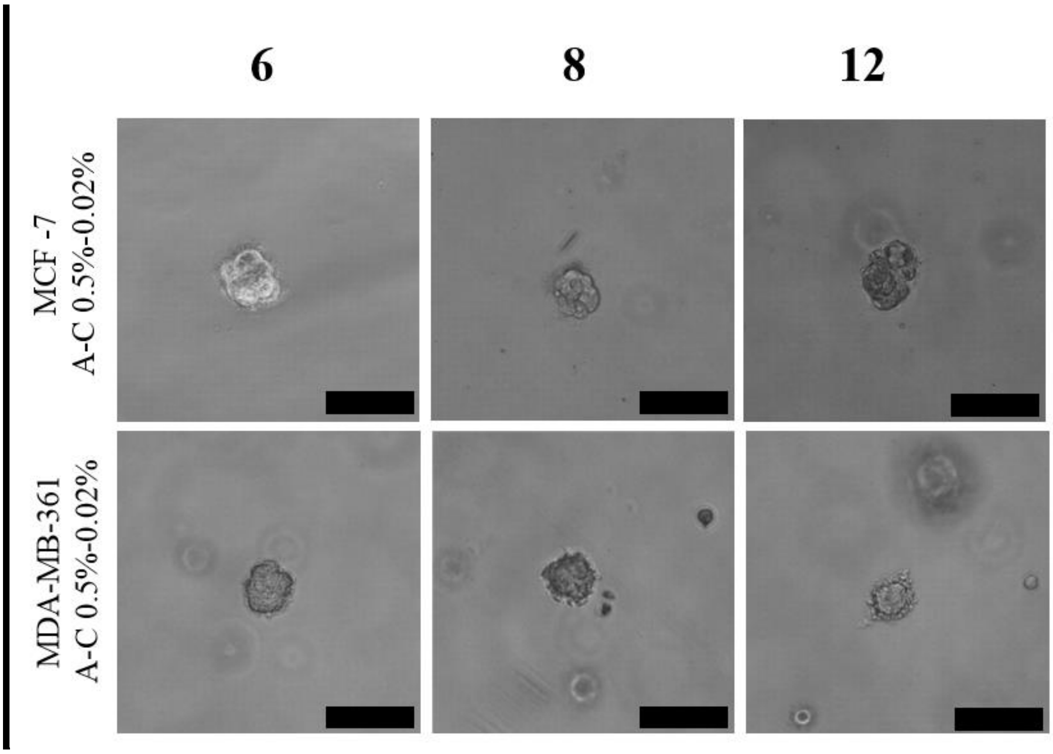

Figure S4. Optical images of the spheroids of MCF-7 and MDA-MB-361 cells grown in the 0.5–0.02% A-C hydrogel after 6, 8, and 12 days. Scale bar is 100 μm.

Table S1. Average size of the tumor spheroids after 12 days of growth in the three types of hydrogels. (*) The size difference between the spheroids grown in the 0.5–0.02% and 0.125–0.02% A-C hydrogels is statistically significant ($p < 0.01$).

	**Average size and shape of the spheroids after 12 days growth**	
A-C Hydrogel	*MCF-7*	*MDA-MB-361*
0.5%-0.02%	64.3 ± 6.5 (spherical)	63.1 ± 7.8 (spherical)
0.25%-0.02%	70.2 ± 8.7 (spherical) *	81.3 ± 6.1 (spherical) *
0.125%-0.02%	91.4 ± 10.8 (irregular)	94.7 ± 9.5 (spherical)

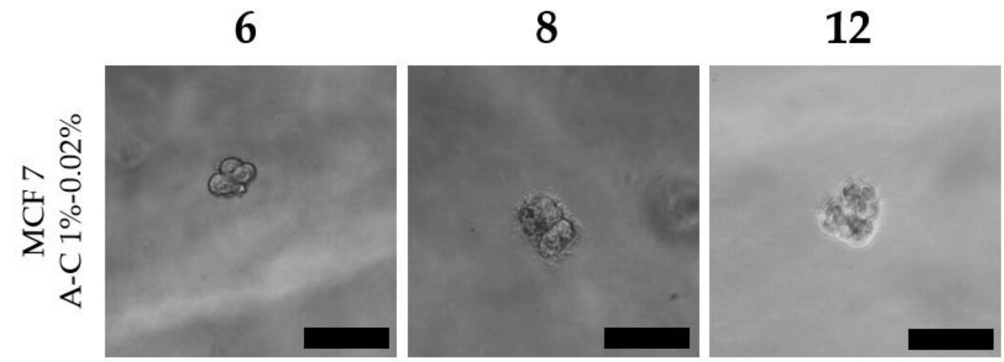

Figure S5. Optical images of MCF-7 spheroids grown in the 1–0.02% A-C hydrogel after 6, 8, and 12 days. Scale bar is 100 μm.

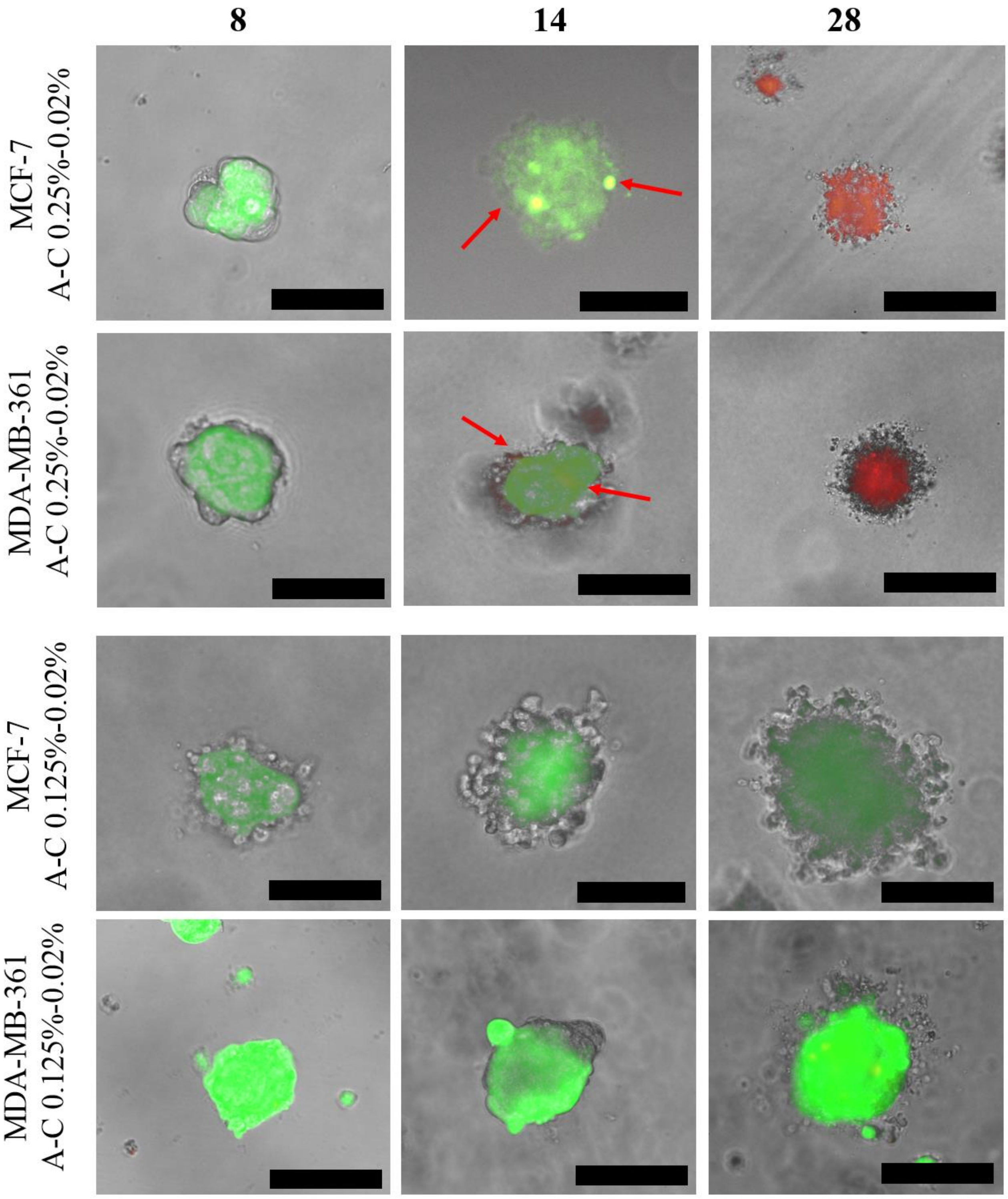

Figure S6. Live/dead assay performed with the cellular spheroids embedded into the A-C hydrogels at 8, 14, and 28 days. The spheroids were prepared with either MCF-7 or MDA-MB-361 cells in 0.25–0.02% and 0.125–0.02% A-C hydrogels. The red arrows indicate dead cells. Scale bar is 100 μm.

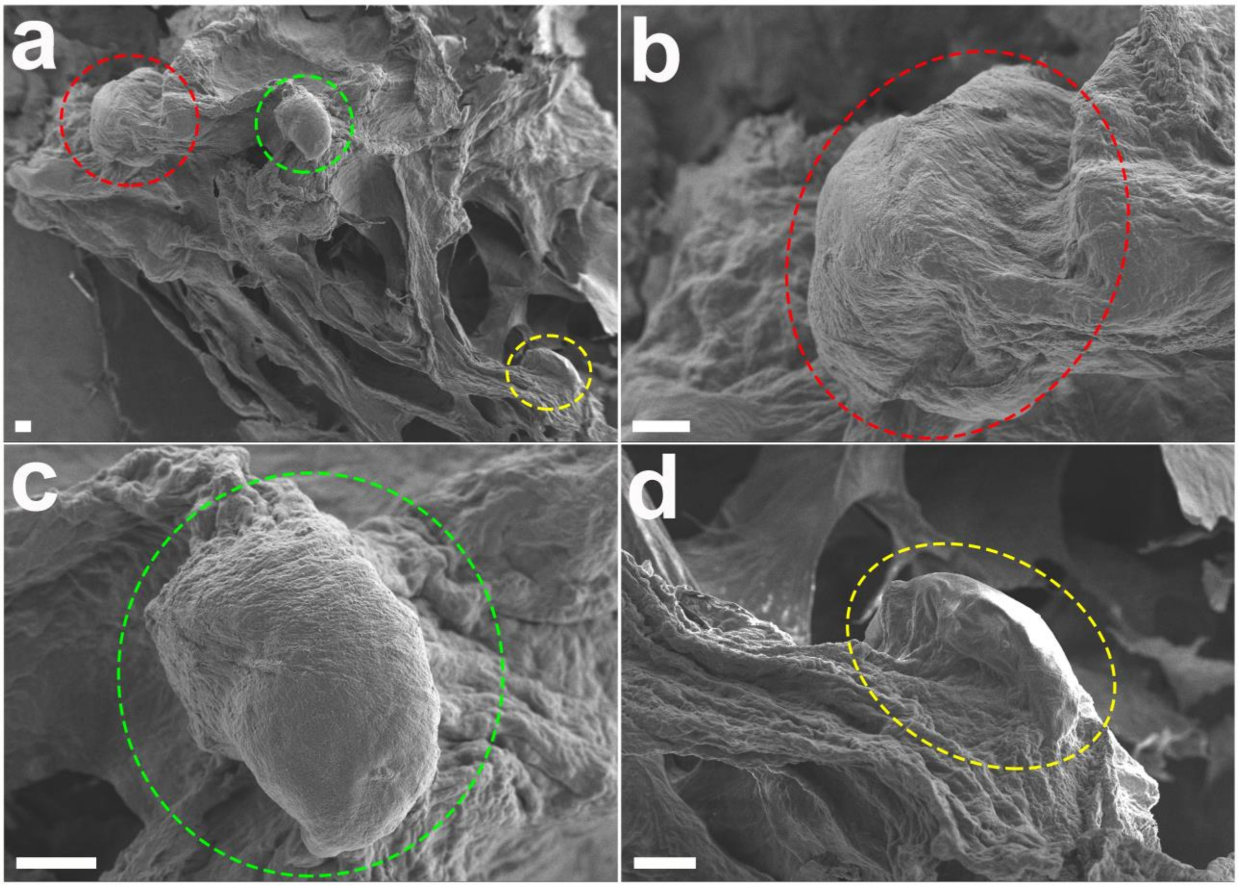

Figure S7. SEM images of MCF-7 spheroids embedded in the 0.25–0.02% A-C hydrogel after 12 days of growth. (**b**), (**c**) and (**d**) are images at higher magnification of the spheroid regions highlighted in (**a**) with the red, green, and yellow dotted circles, respectively. Scale bar is 100 μm.

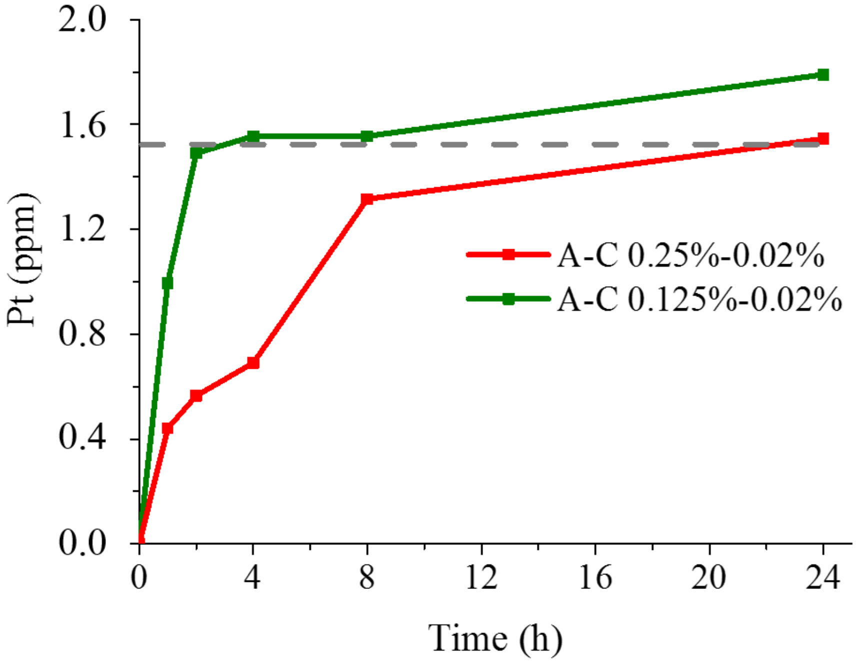

Figure S8. Cisplatin diffusion in 0.25–0.02% and 0.125–0.02% A-C hydrogels estimated after 1, 2, 4, 8, and 24 h incubation with 100 μM cisplatin solution at 37 °C via elemental analysis. Dashed grey line corresponds to the cisplatin concentration at the equilibrium.

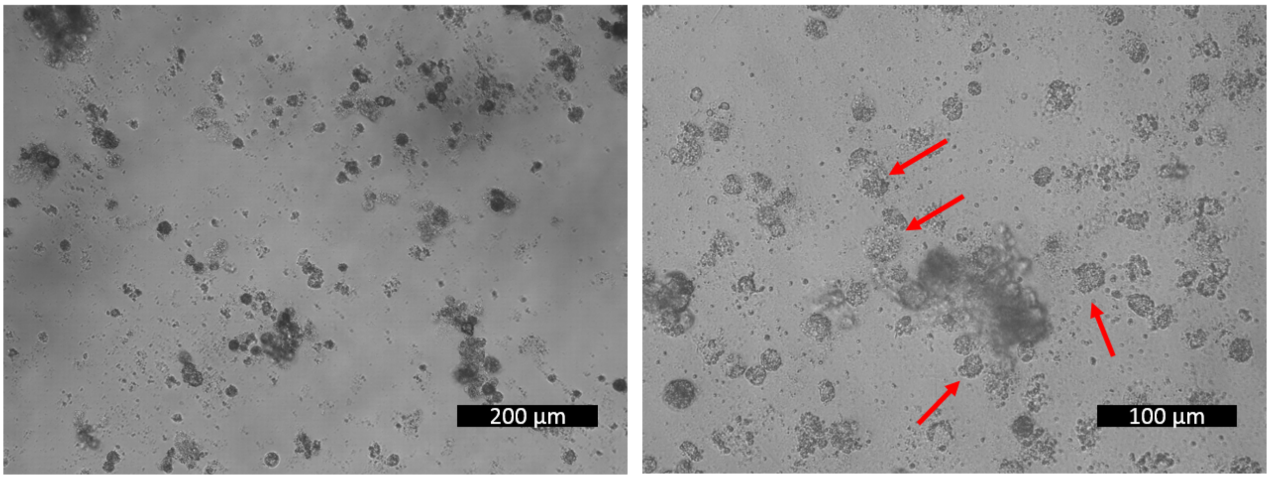

Figure S9. Optical images of MCF-7 spheroids grown in 0.125–0.02% A-C hydrogel after 24 h cisplatin treatment and additional 5 days of incubation with fresh medium. The red arrows indicate the dead cells peeled off from the surface of the spheroids and spread into the hydrogel.

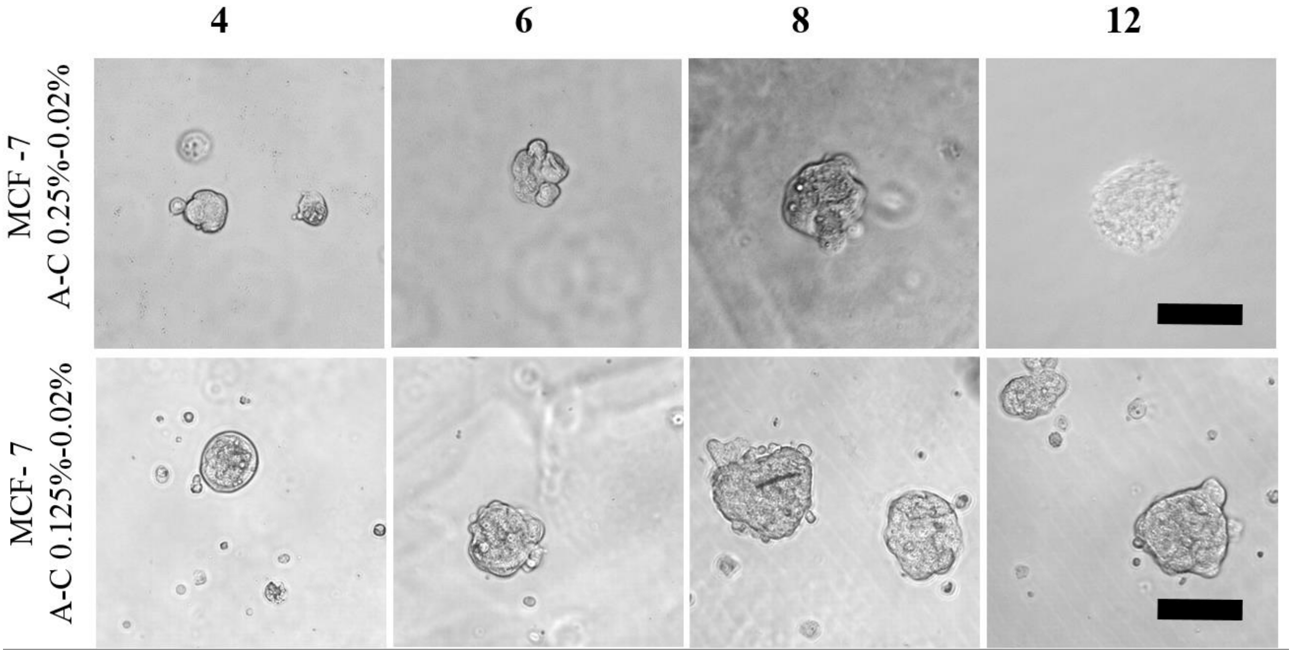

Figure S10. Optical images of MCF-7 spheroids grown in 0.25–0.02% (upper panels) and 0.125–0.02% A-C matrices (lower panels) and recovered after incubation of the hydrogels with agarase. The treatment with agarase was performed after 4, 6, 8, and 12 days of growth. Scale bare is 100 µm.

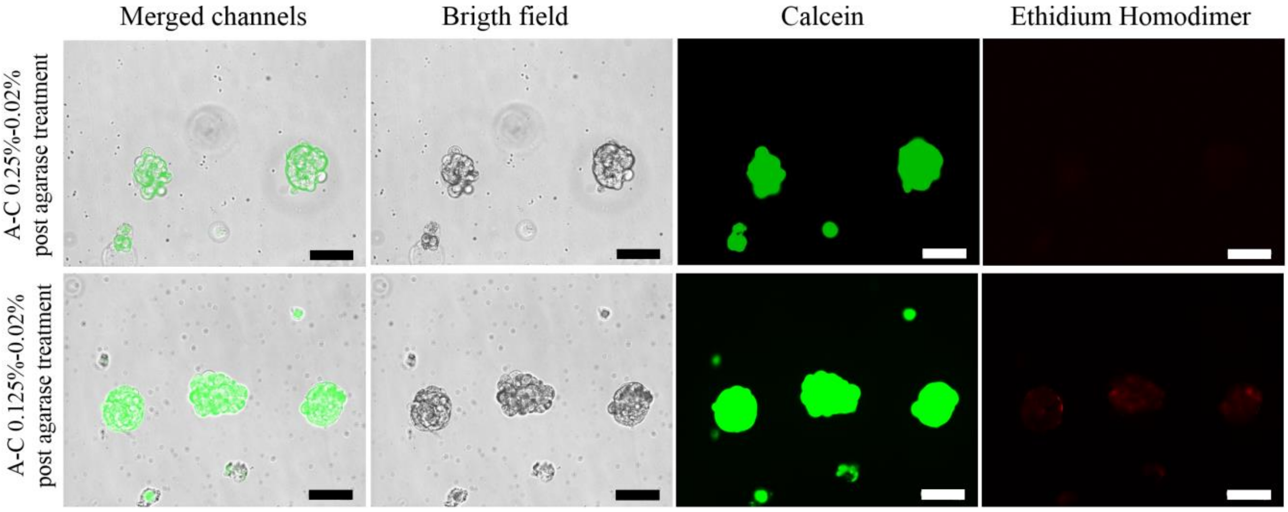

Figure S11. Live/dead assay performed on the MCF-7 spheroids grown for 8 days in the 0.25–0.02% and 0.125–0.02% A-C hydrogels and recovered after agarase treatment. Scale bare is 100 µm.

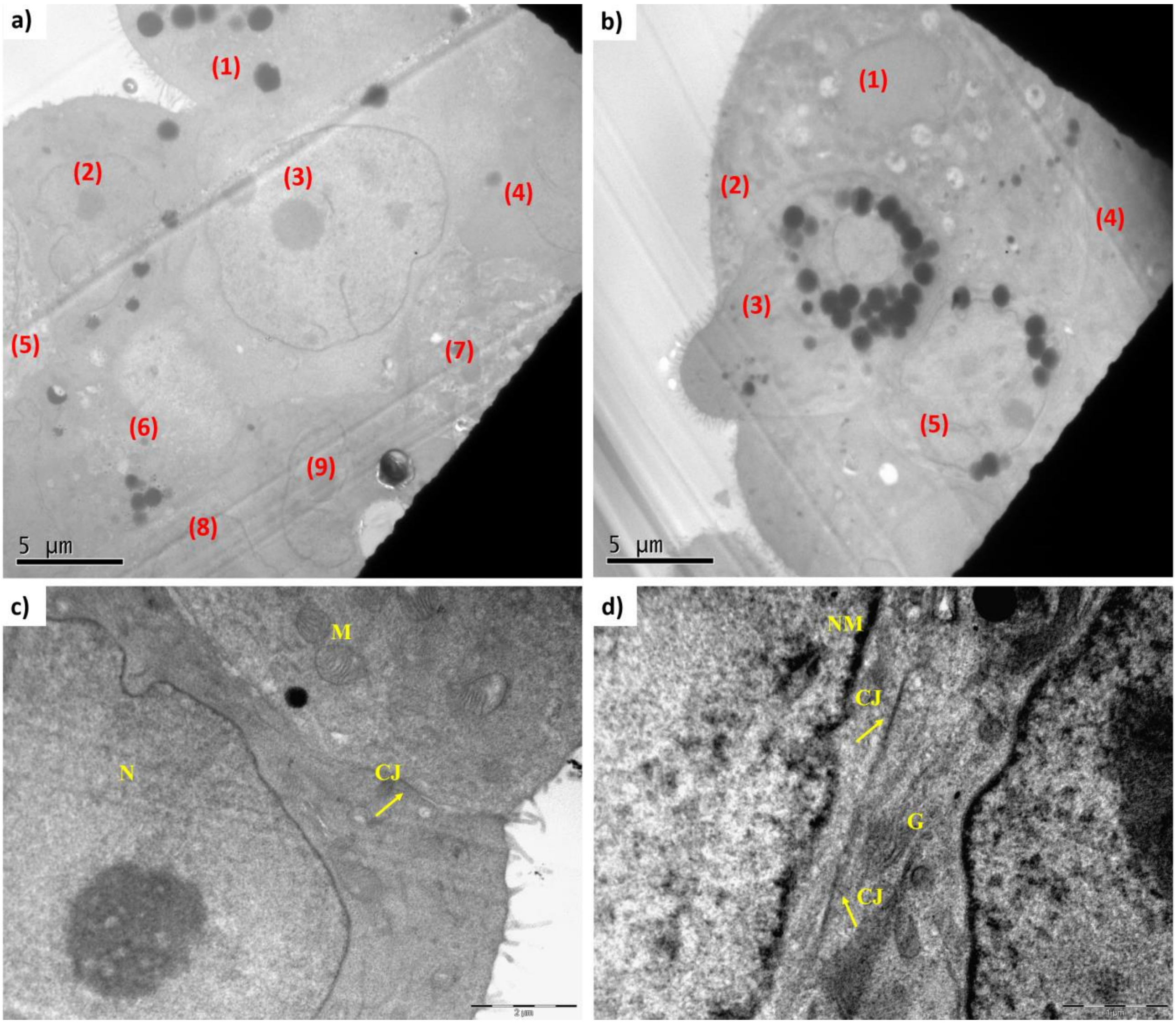

Figure S12. (**a**–**d**) TEM images of MCF-7 spheroids grown for 8 days in the 0.125–0.02% A-C hydrogel. The red numbers show 9 neighbouring cells of a spheroid in (**a**) and 5 in (**b**). The yellow arrows in (**c**) and (**d**) indicate the cellular junctions. The scale bar is 5 µm in (**a**) and (**b**), 2 µm in (**c**), and 1 µm in (**d**). (M: mitochondria; NM: nuclear membrane; G: Golgi apparatus; CJ: cellular junctions).

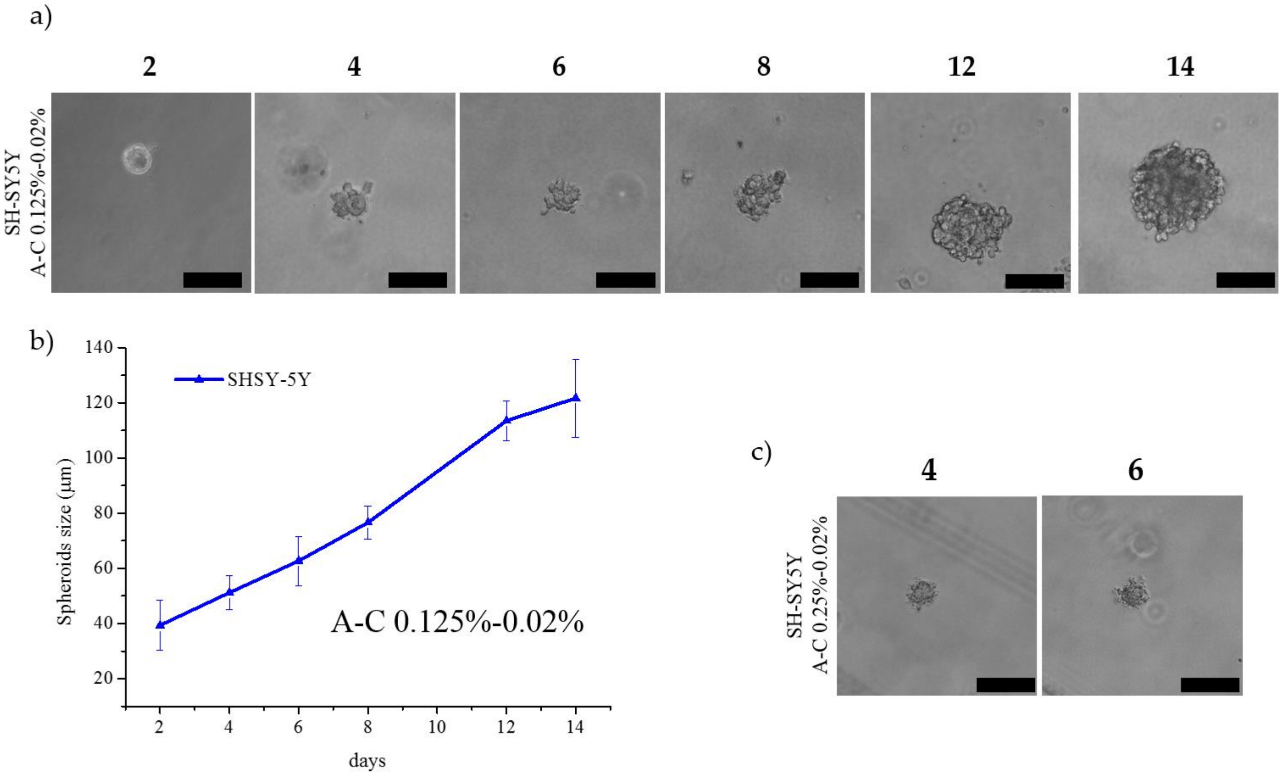

Figure S13. (**a**) Optical images of SH-SY5Y spheroids grown in the 0.125–0.02% A-C hydrogel from 2 to 14 days. (**b**) Growth trend of SH-SY5Y spheroids over time. (**c**) Optical images of SH-SY5Y spheroids grown in 0.25–0.02% A-C hydrogels up to 6 days. Scale bar is 100 µm.

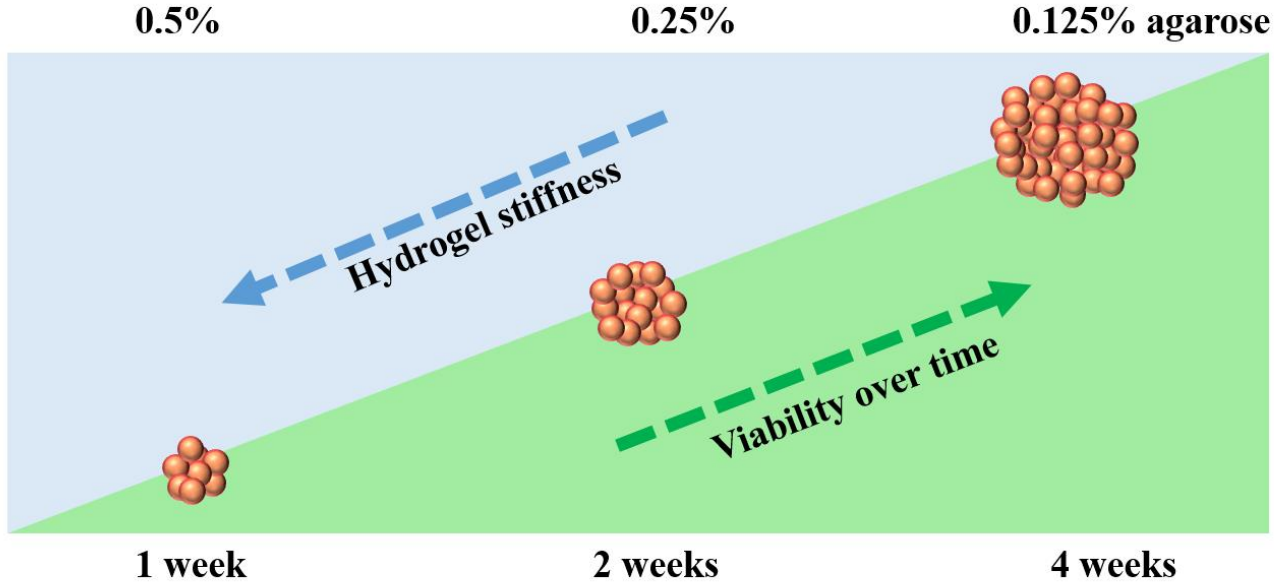

Figure S14. Sketch depicting the effects over time of the hydrogel stiffness on the size and viability of breast tumour spheroids derived from MCF-7 and MDA-MB-361 cells.
